# Women in mid-life and older age in recovery from illicit drug use: connecting and belonging

**DOI:** 10.3389/fpsyt.2023.1221500

**Published:** 2023-08-10

**Authors:** April Shaw

**Affiliations:** Salvation Army Centre for Addiction Services and Research, Faculty of Social Science, University of Stirling, Stirling, United Kingdom

**Keywords:** illicit drug use, recovery, women in mid-life and older age, family relationships, connecting and belonging

## Abstract

**Background:**

Establishing and maintaining healthy social connections and relationships are important in encouraging a sense of belonging that can help mid-life and older aged women in recovery from illicit drug use. This paper contributes to an under-researched area of substance use recovery among women in mid-life and older age by asking what influence social relationships have on their sense of self as they age into recovery from illicit drugs.

**Methods:**

In-depth qualitative interviews were undertaken with 19 women in the United Kingdom who self-identified as ‘in recovery’ from illicit drug use. The interviews were transcribed verbatim and analyzed using Braun and Clarke’s thematic analysis techniques. The study received ethical approval from the University of Glasgow.

**Results:**

As their drug use progressed, the women experienced feelings of estrangement and separation from others. Entering and maintaining a healthy recovery from drug use required the women to break their connections to people considered disruptive or challenging. Creating and setting boundaries enabled some of the women to gain a sense of control over their relationships and recovery. Positive reinforcement from others was pivotal to the process of the women’s self-acceptance, contributing to better self-concepts that helped them maintain their recovery.

**Discussion:**

This investigation into substance use recovery among women in mid-life and older age offers new insights into the relationship challenges they face. It offers suggestions for further research that could support the development of family support programs for mid-life and older age women in active drug use or recovery.

## Introduction

The term ‘recovery’ In relation to drug use, is an imprecise concept (1). As Neale et al. suggest, recovery is not just about ‘taking or not taking drugs’ as people may self-identify as in recovery but continue to use illicit substances (2: p. 15). Recovery is also defined as individuals achieving and sustaining improvements in relationships, health, employment and other areas of their personal (and public) lives (2). It is also argued that recovery is a personal process that defies predictive rules (3). This paper adopts a definition that encompasses the Scottish Government’s characterization as a ‘process through which an individual is enabled to move on from their problem drug use, towards a drug-free life as an active and contributing member of society’ (4: p. 23). As the findings in this paper will demonstrate recovery is a highly personal concept among women who have lived experience of unmanageable drug use.

Self-in-relation theory recognizes that people experience their self in relation to family members, friends, children, neighbors, colleagues and others (5). As ageing occurs, people are prone to experience the loss of key relationships and social networks while the biological and social aspects of ageing can increase the sense of diminished self-worth among older people, particularly among older people who use drugs (6). For women in mid-life and older increasing isolation and marginalization within drug-using circles is exacerbated by disengagement from relationships through the death of drug-using friends, friends who use drugs and enter recovery and stop using, or age-segregation within the drug using environment (6). Nevertheless, a shrinking network of drug-using associates and significant others can be beneficial to reducing and abstaining from problematic drug use and shifting to a new social identity that engenders a sense of belonging (7). Bellaert et al.’s qualitative study of 30 women revealed supportive social and structural factors were needed to create a positive separation from drug-using networks and to increase feelings of connectedness (7: p. 298). However, network members can also provide support which can make it difficult to fully disconnect from relationships that might risk recovery (8). Furthermore, as women age their attitudes and responsibilities toward others may change. For example, Hamilton and Grella (9) study of older people who used heroin found women were more expressive than men about the impact of their drug use on their families and in particular expressed regret and guilt over neglecting their children (9). Similarly, Jessup et al. (10) describe how parenting and grandparenting is a primary motivation to stop drug use but also how reconciling relationships with adult children can be fragile and challenging (10). Interpersonal relationships can be both a source of support and potential triggers for relapse.

For some women, relationships with their own mothers can be challenging but also in many cases a vital source of emotional and practical support (11). In Strauss and Falkin’s mixed method study of 100 women around one-third reported difficult relationships and feelings of abandonment. While some of the women’s mothers provided unconditional support, others encouraged criminal activity and drug use. A breakdown in trust between mothers and daughters was reported with some indicating their mothers exercised control over them or provided unwanted help. These actions made it difficult for daughters to view these attempts at help as supportive. The grandparent role, which usually occurs later in life, is a role through which some women attempt to re-establish their relationships with children and restore their identities as ‘nurturing’ and ‘caring’ women (12). Caregiving for family members can reinforce abstinence or reduce drug and alcohol intake (10) while multiple responsibilities and obligations to ageing parents, long-term partners, children and grandchildren can lead some older women to subordinate their needs to those of others (13). Women’s sense of self is tied to the relationships they nurture or abandon as they move through life. As Guerrero et al. have shown, developing and maintaining empowering relationships are important for women in recovery as they can support and encourage improvements in psychological and physical well-being (14). The importance of social interactions and relationships has the potential to encourage connectedness and a sense of belonging to something that helps shape an individual’s recovery (15). Connecting with others, making and maintaining healthy social relationships are key factors in many people’s successful recovery from drug use.

Belonging as a concept is defined by Vanessa May as a ‘sense of ease with oneself and one’s surroundings’ (16: p. 368). An individual’s sense of self is constructed in their interactions with others and in the abstract notion of collectively held social norms, values, and customs (17). People in recovery from illicit drug use are often described as having gone through some kind of identity transition or change (3). According to McIntosh (18), some are said to revert to an ‘unspoiled’ identity (18) as if being a drug user equates to having a spoiled identity. Identity change in recovery is socially negotiated through a process of social learning and control that is communicated through an individual’s social recovery networks (19). According to Best et al. important to this shift in identity from ‘drug user’ to ‘person in recovery’ is a sense of belonging, support, efficacy and meaning (19: p. 115). As social creatures, a person’s sense of belonging is usually associated with connections to something, someone, or somewhere. Human connectedness occurs when people actively engage with others, activities, objects, or environments that results in a sense of well-being and belonging (20). However, feeling connected and feeling that one belongs are not necessarily contiguous. A sense of belonging requires some form of connection therefore connectedness can be a precursor to, and reinforce, belonging (21). Yet one can be connected but not feel a sense of belonging or have a sense of belonging but not feel connected (10). Belonging involves emotional attachment, a ‘feeling of being home and safe’ (22: p. 647). People perform connectedness and belonging throughout their lives - moving in and out of different places, spaces, and relationships, connecting and disconnecting as they go (23). The individual’s sense of self, the way they see themselves and the ways in which they perform to others, are socially negotiated.

A recent study of 88 women in recovery described ‘relationship actions’ such as disconnecting or limiting contact with recovery-endangering people whilst adding recovery-supportive individuals to help maintain recovery (24). Work by Gunn and Samuels has shown that while some family relationships can promote and support recovery, others can impede recovery through stigmatizing actions and unrealistic expectations (25). This is supported by the work of Sanders (26) who in a mixed-method study of 92 women attending Narcotics Anonymous in the United States, found that women often experienced stigmatizing behaviors within the family and many felt shame regarding relationships that had broken down (26). Families can be sources of trauma and pain as well as love and belonging (27). Belonging therefore has a temporal and spatial element that changes over time, ‘partly in response to changes in our self’, but also to changes in people and the world around us (16: p. 372). Belonging is a dynamic practice that is temporal in nature. And so too is recovery. People move into, through and beyond recovery. Eventually, recovery can belong to an individual’s past. As a sense of self changes, so does a sense of belonging, and as a sense of belonging changes, so does a sense of self. Belonging is therefore a multidimensional experience in which people experience multiple senses of belonging across time and place. Women who engage in a mix of meaningful and multiple activities are likely to develop identities beyond that of women ‘in recovery’ or ‘ex-drug user’ (28).

In this paper, the research question, what influence do social relationships have on women’s sense of self as they age into drugs recovery? is explored through an analysis of 19 qualitative interviews with women in mid-life and older with histories of using illicit drugs and other substances. The phrase ‘age into drugs recovery’ refers to the temporal aspect of moving from drug use to (self-defined) recovery. The women in this study were interviewed at a single point in time but they reflected on their lives from childhood through to where they are now and oftentimes their hopes and aspirations for the future. It was considered appropriate to use the terms ‘age into’ or ‘ageing into’ to describe the dynamic and temporal process of the women’s recovery journeys. For the purposes of this study, ‘older’ in the context of problem drug use was defined as aged 35 years and older in line with published Scottish and European research (29, 30). This cut-off may seem young but long-term drug use is likely to accelerate the ageing process and its accompanying conditions, with some authors suggesting long-term drug users who start at a younger age may have a biological age some 15 years older than their chronological age (30, 31).

While there is a growing body of academic research exploring the effects of drug use and the treatment needs of older people who use drugs, there remains comparatively less work exploring the needs of women in mid-life and older who use drugs or are in recovery from unmanageable illicit drug use. In light of this absence of research in the field of drug use and recovery, the decision to look at the specific issues surrounding women in mid-life and older was considered appropriate and relevant to understanding the issues that affect them at this stage in their life.

## Methods

### Methodological approach

The methodological approach of this study is grounded in an interpretivist and feminist paradigm, namely symbolic interactionism (32) and feminist standpoint theory (33). Symbolic interactionism is based on three underlying principles. Firstly, people act toward things (for example objects, institutions or guiding values) based on the meanings the things have for them as individuals. Secondly, meanings are derived from the social interactions people have with each other. Thirdly, meanings are managed and revised through an interpretive process used by individuals in dealing with the things they encounter (32). Accordingly, symbolic interactionism views meanings as ‘social products’ that are formed through the activities of people as they interact (32: p. 5). In other words, people’s perception of who they are in relation to others and the social systems in which they live is worked out through their interactions with others. Feminist standpoint theory further shapes the methodological approach of this study. Feminist standpoint theory is an interpretivist approach that provides a methodology for understanding ‘relations of power as a distinctive kind of obstacle to the production of scientific knowledge’ (33: p. 219). One strand of standpoint theory holds that people who are marginalized or otherwise unheard are epistemically advantaged in understanding their position (34, 35). In other words, they are critically conscious and aware of their social position (35). Wylie counters criticisms that feminist standpoint theory is essentialist or individualistic by stating that women’s position and understanding do not necessarily mean they have ‘epistemic privilege of how or why their oppression originated or is maintained’ but they do have an alternative knowledge and understanding that can be compared to the dominant worldview (34: p. 37). Feminist methods have traditionally taken a qualitative approach to data collection, rejecting the positivist approaches that emphasize objectivity and detachment, arguing instead for a more egalitarian, open and active process between researcher and participant (36, 37).

### Recruitment

The study was advertised via postings on online recovery sites, on flyers and posters in recovery cafes, and through email distribution to colleagues and contacts who forwarded on to potential participants. Women who voluntarily chose to participate were included if they met the following criteria: identified as a woman, aged 35 or older who had a history of illicit drug use and self-identified as being in recovery from drug use (abstinent or low risk use). Individuals were excluded if they did not meet the inclusion criteria or self-identified as having mental ill health or other issues that might trigger distress during the interview or were non-English speakers. As the women contacted the researcher directly it was not possible to ascertain mental health status other than via their own self-assessment. However, the women were given information about the study and topics for discussion at first contact. They were also asked prior to the interview if they were happy to proceed and if there was anything that might cause them distress during the interviews. No-one declined to be interviewed, no-one indicated distress, and no-one withdrew prior to, during or after their interview. Non-English speakers were excluded due to costs incurred hiring translators.

Convenience sampling was used to ensure enough women were recruited within the time available and to avoid delays to fieldwork. As this was an exploratory study, it was not deemed necessary to undertake probability sampling. For example, there was no intention of analyzing data by characteristics such as race, social class, or sexual orientation. There was an attempt to ensure the views of women from different geographical areas were included to minimize the clustering of participants from predominantly urban areas in Scotland. Recruitment for studies that explore the views of people with lived experience of using drugs in the United Kingdom frequently focus on treatment and support services in urban areas, consequently, the voices of women from rural areas are heard less often (38, 39) hence the effort to include them in this study. Voluntary and other non-statutory organizations working with women who have used drugs were contacted in coastal towns on the east coast of Scotland and towns in the far north and south of Scotland. All these areas are rural or semi-rural. Recruiting from these areas met with varying degrees of success and therefore, women were recruited from the North, West and East of Scotland and the North-East England from a range of rural, semi-rural and urban areas.

### Topic guide and interviews

The topic guide was piloted and developed by the author, as part of their Master’s thesis, with nine women from Scotland in mid-life and older age who identified as in recovery from illicit drug use (40). The advantages of piloting the topic guide was that it helped hone the questions, identified challenges in the interview process, and enabled the identification of follow up areas of further interest. The topic guide is available on request from the author. All the interviews were carried out by the author, (at time of interviews) a 50-year-old woman with 17 years’ experience in the field of substance use research, working in both academic and third sector (not-for-profit) settings.

### Analysis

An inductive analytical approach was used in this study. This approach develops concepts and themes from the data and is an iterative process whereby the data are collected and analyzed simultaneously (41). The interviews ranged in length between 68 and 182 min (mean average 131 min) and were transcribed verbatim by the author. To ensure their accuracy, a sample of five transcripts were checked against the recorded data for quality control and seventeen participants were given the opportunity to read through their transcripts. Two participants did not have an opportunity to check their transcript as their contact was through a third person. Maintaining the women’s confidentiality was crucial and as such their transcripts were not forwarded. Of the 17 who were given the opportunity to read and check their transcripts, 12 took up the offer. No participants raised any issues, corrections or complaints.

The transcripts were analyzed using Braun and Clarke’s thematic analysis technique (42). The qualitative software package NVivo 11© was used to code and categorize the data. The interview transcripts were coded through six phases: familiarization with the data, transcription, initial coding, searching for themes, reviewing themes, defining themes, and report writing. Transcribed interviews were read through at least twice for familiarization and a coding framework was developed as interviews progressed to identify themes and sub-themes from the interviews. Borrowing from grounded theory (43), open and selective coding was utilized to provide a systematic approach to the development of themes. Selective codes were developed based on verbs instead of nouns thereby focusing attention on the social processes in the data (44). An example of open and select coding under the thematic code ‘relationships’ is shown in Table 1. Several top-level themes were already identified from the pilot work and related to the study aims. For example, *a priori* themes included relationships, ageing, and recovery. The coding was further refined as interviews progressed and with repeated reading of the transcripts. The author maintained a coding book, in which codes, descriptions, and examples were detailed and discussed with the author’s PhD supervisors. No discrepancies arose between the PhD supervisors and author as the codes and descriptions were presented. Supervision meetings were monthly, and both provided informed support throughout the entire period of the PhD, including through the fieldwork and analytical stages. The author is an experienced qualitative researcher with extensive coding experience and both PhD supervisors are highly experienced and published female researchers and academics in the area of addiction and recovery.

**Table 1:** Open and Selective coding examples.

Theme		Open coding	Selective coding
Relationships	…when your home life is just as bad as at school then I think that’s why I first started taking amphetamines because it was like ‘yeah’. Instead of being miserable all my life it was like ‘yeah this is a happy feeling.	Childhood School Family dynamics Drugs_sense of belonging Looking back_remembering	Belonging
	And I do find it quite easy to cut people out now, it took a long time but no I will not take it. I think that’s maturity. Maturity.	Breaking social bonds Ageing_Older Control_in control	Disconnecting Ageing
	My other brother has just served 5 years for drug dealing. Hence why I live 200 miles away from my family. Because I work in [*criminal justice post*] I cannot associate with that type of person. And I think that’s one of the biggest things I’ve learnt is that a lot of people from my past, I’ve kind of had to leave them there.	Breaking family bonds Breaking social bonds Siblings Identity Changing roles & responsibilities Recovery_challenges Employment	Disconnecting from family
	If I think about all the money I spent and what I could’ve done but it’s gone now so it’s just get on with it. Yeah do not sit and get upset and go ‘I could’ve done’. It’s gone. That’s it. So yeah everything else is just building. Me and my mum got a good relationship now and pretty much everybody I talk to now I’ve got a good relationship whereas before I just didnae bother with anybody.	Looking back_remembering Recovery_work in progress Building_Rebuilding Mother Isolation Connectedness_belonging	Building Reconnecting
	I think that’s maybe why I was nervous. Probably starting to come to the recovery cafes because I thought it’s all going to be teenagers and young people. And I think that’s what older women think. ‘oh it’s not for me. It’s not for me its for younger people.’ You know. And we have such a laugh when we are making jewellery and things like that. You know some of the women they have got amazing stories and they are so… like… …well like they are different to guys aren’t they. They’re so supportive of each other like really genuine.	Recovery Making connections Stigma_felt Bonding capital Women as support Ageing_Older Connectedness_belonging	Connecting Belonging Building Ageing

### Ethical considerations

Women who expressed an interest in the study were given a participant information sheet and at least 2 days to consider whether to take part before signing a consent form. The study was approved by the University of Glasgow’s College of Social Sciences Research Ethics Committee (application no: 400170200). The women were assured complete anonymity and all names used in this and other papers are pseudonyms.

## Results

### Sample

Nineteen women with a history of using illicit drugs and in self-defined recovery participated in the study. The women were aged between 36 and 60 years (mean average age 47) and resided in a mix of urban (*N* = 10), rural (*N* = 6), and coastal locations (*N* = 3) across the North of England and Scotland. The participants used a range of drugs including heroin, powder cocaine, crack-cocaine, and cannabis for between 7 and 47 years (mean average 21 years). The age at which the women self-reported stopping illicit drug use ranged between 26 years and 54 years of age (mean average 34 years of age). The women’s recovery time was self-reported and ranged from 6 months to 18 years (mean average 9 years). At time of interview, 16 women reported no drug use and three women reported occasional low-risk drug use, including intermittent use of cocaine, cannabis, alcohol, amphetamines and heroin (low-risk is defined as illegal drug use at minimum level causing no psychological, legal, employment, family or health problems) (45: p. 83). Eighteen women had children (ranging from a months old baby to adult children). Just over half (*N* = 10) reported being in an intimate partner relationship at time of interview, the remainder were single. Though not asked, five women described their backgrounds as working class (*N* = 3) or middle-class (*N* = 2). At interview, nine women were in paid employment, seven volunteered their time and skills (five of those in recovery groups), one attended college, two were not in employment. Seven women had spent time in prison, none were currently involved in the criminal justice system. No details on sexual orientation, income or education levels were asked for or given. All the women were white Scottish or English.

### Remembering and reimagining belonging

During childhood and adolescence, young people come to know the social groups to which they belong and wish to connect to through various forms of socialization (46). Gillian (aged 40, recovery 10 years), said of her early heroin use*: “I thought, this is what I want to do every day for the rest of my life. Now I know that I belong somewhere.”* Gillian’s sense of belonging to a drug culture gave her a sense of purpose in life*: “It was the first time that I thought, this is what life’s about. I felt me calling in life.”* Like many of the women in this study, Gillian’s early memories of a sense of self was as the ‘*outsider’*. Other terms the participants used to describe themselves when they were children and adolescents were: ‘*a fuck-up,’ ‘not part of things’, ‘scapegoat’, ‘never felt valued, always felt like the oddball,’* and *‘a kinda weird kid’.* These feelings and identities as oddballs and misfits were felt as adults too. For many, their memories of themselves as children and sense of their place in the world resurfaced during the interviews, or as Lorna described them, were ‘*reimagined’* in adulthood. Remembering family life as children, some of the women spoke about experiencing domestic violence in the home, of a sense of not belonging and for a few, time spent as a looked-after child in foster care or children’s homes. Leaving home was a source of escape from the fractious family life they had experienced. As adult women, managing these family relationships in a healthy way were important. For some of the women, the sense of alienation felt as an adult was a continuation of the sense of alienation felt as a child and adolescent.

### Disconnecting from the social

As noted in the introduction, some people who use drugs experience a sense of alienation from significant others and the wider social world (6, 18). The women in this study experienced such feelings of estrangement and separation from others:

*“People’s attitudes… when you become a non-person. Where you become your disease. Where you become just a junkie. You become that label, you know, and that really rubs with me because it is again that rejection. Rejected by society because I'm not conforming to the certain standards that you require me to conform to because I'm suffering and we don’t want suffering people in society”* (Lorna, age 53, recovery 18 years).

Lorna’s feelings of rejection from society underpinned her sense of being an *outsider* and a non-conformist. Claire, aged 39 and in recovery for 18 months, decided to finally quit after using drugs for two decades when she realized there was ‘*nothing and nobody left’* to help her. For some of the women interviewed, it was the disconnection and isolation brought about by their use of drugs that encouraged their move into abstinence and recovery. However, leaving behind the drug-using settings and culture was challenging for others. Ruth, for example, spoke about the sense of loneliness she felt in the early days of her recovery and how she returned to her drug-using networks to reduce the social isolation she experienced:

*“I would like go to [Narcotics Anonymous] meetings. I would just … I would see people there and then I’d come home and I’d be in by myself and then you can only do that for so long, like the fear of creating new relationships with people. And then coming home and feeling isolated. There was that in-between bit so I ended up the fear of starting new relationships was too much so I just turned back to people I knew because the loneliness was kinda getting to me”* (Ruth, age 36; recovery, 10 years).

Ruth was learning, in the *in-between,* to deal with her fear of developing new relationships without using drugs. Going to Alcoholics Anonymous meetings was ‘*scary’* while communicating with people was difficult, *‘I could not look anybody in the eye, if somebody spoke to me I got all flustered… I really struggled…*’ Alleviating the loneliness she felt at this stage in her early recovery, Ruth returned to the people she knew and felt comfortable with: *‘the same people that I’d used with.’* Working on her confidence and self-esteem was crucial to Ruth’s recovery, not only from drug use but from the associated lifestyle and intimate relationships that accompanied it:

*“I still felt like I wasn’t worth anything, I didn’t deserve a good life, sort of thing. I was still attracted to people who didn’t treat me right. But in the last three years that’s really changed because I realized I was still making the same mistakes and I realized I was headed back down that path of picking up drugs again and I had to really look at why that was and it was because I was still living in the chaos… I found it really hard to build relationships with people and that was just really like I had really low self-worth and really low self-esteem and I didn’t think I was good enough for people to like”* (Ruth, age 36; recovery, 10 years).

Feeling undeserving and unvalued, Ruth’s ability to make connections and build new relationships was difficult and took time to develop. It was only after 7 years into her recovery that Ruth felt able to move on from the drug-using networks she had stayed connected to while abstinent. Ruth’s example clearly shows the precarious nature of recovery for some women. Moving from the familiar environment of drug use with all its networks, connections, and routines, to the unknown territory of abstinence, recovery, and fellowships (such as Alcoholics Anonymous, Narcotics Anonymous and Cocaine Anonymous) is daunting. Returning to environments that are familiar, even if they carry the threat of danger, might be preferable to being isolated and feeling alone, especially in early recovery.

Levels of social and economic capital can impact on an individual’s ability to preserve or terminate relationships. This is pertinent in drug-using communities where individuals may wish to put physical distance between themselves and drug-using peers but are unable to due to circumstances. Without recourse to alternative housing and with family living close by, Grace’s connections to her relatively recent drug-using past were disrupted and displaced by her social distancing from the local drug scene:

*“The friends you think you’ve had over the years. People coming to your house for coffee and going and taking kids to school and whatever. They just all kind of disappeared when I stopped taking drugs and stopped people coming to my house, when I wasn’t available for their needs. I really thought they were my friends. And I would think to myself I know how they treat other people but thought they wouldn’t treat me like that”* (Grace, age 49, recovery 5 years).

After 5 years in recovery, Grace was still coming to terms with the disruption and disconnection from her social networks. Grace felt a sense of loss and disbelief that the friends she had known for years – shared her home and drugs, confidences, and childcare with - had ‘*disappeared’*. Having lived in the same house for over two decades, she felt a sense of shared history with these former friends. Nevertheless, to connect to and rebuild supportive social networks, the women in this study had to undergo processes of disconnecting, not just to those in drug-using networks but also to those considered no longer helpful for sustaining healthy relationships.

### Disconnecting from the family

Disconnecting from relationships the women considered problematic, unhealthy, and unhelpful was not without its challenges but letting go of long-standing relationships in order to maintain recovery was vital for some. The relationships the women had with their families, and particularly their mothers, were often complicated. The women’s mothers had in some cases experienced trauma such as rape and domestic violence eliciting empathy and sympathy from the women. Nevertheless, maintaining a sense of control in these relationships was difficult for some of the women interviewed. Breaking, losing, or minimizing contact were ways in which the women were able to manage these difficult relationships. Leaving behind the difficult family life they experienced and managing these relationships in a healthy way was important to the women and their recoveries. Evelyn, for example, found new connections and belongings after moving to the United Kingdom mainland. Her sense of belonging was connected to her new partner, his family, their baby and her new flat in a new city. Leaving behind the island where she had lived all her life, her family and the legacy of her drug use was not without some fear: *“Shitting my pants because I’ve always lived on that shitty island but do you want to know something. It’s worked.”* Life had become extremely difficult for Evelyn and her relationship with her mother had broken down to the extent that she did not contact her for over a decade. This strategy of physically and socially distancing herself from her former drug-using environment and negative family influences enabled Evelyn’s recovery: *‘… that worked for me. Because I felt like I was always getting caught up in madness.’* In recovery, Evelyn eventually reconnected with her mother but set boundaries to help her maintain some control in their relationship and her life:

*“What I need to do is boundaries to keep what I've got, know what I mean? I'm going to fight for that because my mum’s, and I don’t mean this in a nasty way, my mum’s back there. My mum’s older right… If I start lowering my boundaries and be like start going to pubs and clubs with my mum then that’s me inviting the devil in”* (Evelyn, aged 38, recovery 3 years).

In her new environment, the support she received from her current partner and his father, in addition to outside support from social services, contributed to Evelyn’s ongoing recovery and her ability to care for her baby. Nevertheless, Evelyn’s re-building of a ‘new’ life meant disconnecting from parts of her past life. She said of the island she had left, *“I had to leave there to get my recovery.”* Of people she had known, they *“serve their purpose, you’ll outgrow them, or they’ll outgrow you or you just do different things on your journey.”* Creating and setting boundaries with her mother and others in her social network enabled Evelyn to gain a sense of control over her recovery and her ability to care for her baby.

Similar to Evelyn, Kerry had a difficult childhood and felt compelled to leave home when she was 16. Having spent years trying to ‘*repair’* the relationship with her sister and mother, she eventually decided to stop doing so. In doing so, her mental health improved:

*“I don’t see my mum or my sister. I haven’t seen them in 11 years. We never got on growing up me and my sister. My mum didn’t care less about what happened to me, that’s what it seemed like. I tried like once I had my kids to try and repair the relationship but no. There’s no point trying to fix something that’s not going to fix is there? It’s just making me more stressed and more upset, and I think I’m better off without that, and once I cut them out my life it was, it was a bit of a relief. I know that sounds awful, but I’ve got my close friends and my family. I’ve got my auntie and I'm happy enough with that”* (Kerry, age 43, recovery 5 years).

Having a supportive partner and network of long-held and trusted friends, Kerry felt able to break the family bonds with her sister and mother. Like Evelyn, discussing her mother raised uncomfortable feelings, particularly around the guilt Kerry felt for feeling the way she did. Kerry said: *“I know that sounds awful,”* while Evelyn stated, “*I do not mean this in a nasty way.”* Relinquishing the obligations women think they have to their mothers is perhaps one of the more difficult relationship transitions women who use drugs or in recovery experience. For example, Janine said of her mother: *“I let her go. I let her go. Ehm _ _ yeah. Big, big taboo thing for a daughter to do, but I had to do it.”* The women internalized what they perceived as the socially normative expectations of how a daughter should act and feel. However, moving into their recovery, these expectations differed from their own perceptions of their needs and desires as women in their own right, rather than the daughters they thought they should be. For some of the women, the tension between the expectations of others and their own aspirations led to feelings of guilt.

The expectations, attitudes, beliefs and values within families and society more generally are important elements of the social context in which recovery is performed (11). And, of course, women are daughters to fathers too. For some women in this study, relationships with their fathers were also challenging. Many had grown up with violent fathers who abused their mothers and sometimes the women themselves as children, and their siblings. Fiona talked about breaking the relationship bond with her father ‘*bit by bit’* and, like Janine, she considered it outside the norm for a daughter to do this to a parent:

*“I’ve always known my dad’s been a violent bully. But how, as a woman, do you stand up to that? How, as a daughter, do you challenge that? And I won’t ever say it was okay, I kind of distance myself from him because I don’t like him. And I refuse to be around people I don’t like. One of my biggest sayings is ‘if you don’t bring joy to my table, there’s no seat for you.’ And I distanced myself little bit by bit”* (Fiona, age 44, recovery 17 years).

Even as women in mid-life with all their life experience, there was a questioning of appropriate and socially acceptable behaviors in relation to challenging parents and disconnecting from the parent–child relationship. These findings are important, for while there is a lot of research exploring women’s relationships with children and intimate partners, there is far less that engages with women’s relationships with their parents and their mothers in particular. Most of the women’s interviews included discussion around their mothers and often how their mothers challenged and undermined the women’s sense of self-worth and self-esteem.

### Building and reconnecting

Building a credible abstinent identity relies on the symbiotic nature of relationships. Abstinent identities are reflected to some degree in how others perceive and believe in the veracity of the performance (28). Reconnecting to people, whether family or the wider world requires work and for some of the women they spoke about this in terms of ‘*building’*: building connections, building self-esteem, building on the work they had already done on themselves. In Claire’s extract, she discussed ‘building’ the relationship with her family. She showed how, in recovery, trust was built up over time, how she was present not just physically but also emotionally when she was with them. Claire recognized the differences between her past and current relationship with her children, and found it emotionally difficult to contemplate:

*“… other important relationships are with my family now. Building on that. It’s taking time to put things right and for them to trust me but I’m just, it’s different now because I’m with them now, I’m with my kids, I’m present with them and a lot more honest with them. I can listen to them a lot more than I did”* (Claire, age 39, recovery 18 months).

Multiple periods of abstinence followed by evermore chaotic behavior engendered a lack of trust and confidence in Claire’s recovery, and this was something she had to work at proving to her children, parents and other family members. The person recovering from drug use needs to prove to others that their abstinent identity is genuine, and this often takes time.

Building relationships with family can be difficult, particularly when there is tension between people’s remembering of them in the past and their expectations of them in the present as shown in Sophie and Maya’s extracts below:

*“Aye and even like building relationships with your family. If it doesnae work, it doesnae work. You cannae keep you know pedaling the bike when the tyres are flat… for a lot of times me being the facilitator of making things right, of doing things, being the carer. That suited everybody in my family and then now that I’m not doing it, somebody else has to do it and eventually they’ve had to become alright with that… it’s like we need to rebalance this because that’s the only way that families can coexist. It needs to be balanced”* (Sophie, age 55, recovery 5 years).

*“I’ll be saying to my older brother soon enough ‘I am not, I am no one’s carer and I don’t want to be. I don’t want to assume that role. I don’t want to take on that role any longer for my mum. My mum’s going to be leaving the care unit, find her somewhere to stay down where you live’ because I spent all of my days being a carer, caring for people and I don’t want it. I don’t want it anymore. I’ve had it imposed on me and I’m in a position now where I can say no, I deserve to have my life and focus on me”* (Maya, age 42, recovery 14 years).

In the past, Sophie’s role was to be the ‘*carer’*, the go-to person in the family. Despite some pushback from family members, Sophie’s refusal to continue in the carer role resulted in a recalibration of her family relationships. Maya was still in the process of renegotiating her role as carer within the family but like Sophie, she was determined to develop a new dynamic in which she would have more control over the demands made on her. Recovery is not just about the actions of the person who is abstaining, it is also about the actions of family members, relinquishing their patterns of behavior toward and expectations of the women’s roles within the family.

Building the foundations for healthy relationships with the self and others is important for maintaining a sense of being valued. Ruth explained how she builds feelings of self-worth incrementally: “*it was just like loads of wee things like that kinda thing that just kinda built me bit by bit.”* Ruth uses the lessons she learned from her evolving relationships in recovery as a foundation for relationships with family and others. In turn, this leads to a better sense of self – one in which she can hold her ‘*head up’* and look people *‘in the eye.’* In other words, one in which she feels equal to others and not less than:

*… that’s kinda rippled out into, that’s the foundation of how like I started, changed my view of relationships and how people should be treated and how you should be treated yourself and I’ve kinda took that and built on it and expanded it into my immediate, like my family. My siblings and my mum. And worked on my relationships with them from that”* (Ruth, age 36, recovery 10 years).

Ruth acknowledges the hard work and time that is required to rebuild relationships and reconnect with family members: *“I had to work really hard. Be really patient … it’s just like chipping away.”* Having put the effort and *hard* work into her recovery, Ruth finally felt validated by her mother:

*“She’s just started saying like how well, how proud she is, how far I’ve came, how well she thinks I’m doing, how different my life is, how different I am as a daughter and a sister, and how she knows she can rely on me and if I say I’m going to do something I do it… that’s the kinda stuff you’ve been chasing your whole life isn’t it? Is for your mum to acknowledge you and give you a bit of praise and tell you that kinda stuff”* (Ruth, age 36, recovery 10 years).

Rebuilding relationships in recovery requires managing the expectations of other people, whether family or wider society. As the women age into their recovery, older parents may create additional caring responsibilities that women in drug use recovery will have to address and negotiate, potentially with other family members.

### Connecting and belonging

Recovery communities play a seminal role in nurturing and developing a sense of connectedness and belonging. They can offer a ‘collectivity’ through shared meanings and experiences and offer resources and moral guidance to help members follow an alternative life-path (47). Although most had engaged with recovery communities and fellowships at some point in their recovery, those in early recovery (<5 years) were more likely to report connections to other women in recovery whereas the women in long-term, stable recovery were more likely to report a wider field of connections beyond other recovering individuals. Entering new spaces and locations, building, and maintaining new relationships requires individuals to learn new habits, new ways of communicating and new ways of being. This ‘boundary crossing’, (47: p. 22) if successful, enables individuals to acquire new knowledge and experiences that can help empower them to cross other social boundaries. As Janine’s experience demonstrates, recovery communities are one element in which women who have used drugs can cross boundaries of connection and belonging:

*“… where I’m from a working-class town, you don’t get to meet you know _ people from other well you know what it’s like in the UK, its different classes, different educations, different countries. Because of the 12-step program, AA, NA, I’ve met people from all over the world. Gained different perspectives, different viewpoints. It’s took me out of that West coast of Scotland, Irish catholic mentality and opened my mind…”* (Janine, age 47, recovery 21 years).

For Janine and some of the other women, recovery communities offered opportunities for wider social participation. Within and out with the fellowships the women met and socialized with people from different social classes and backgrounds, learned new ways of being and further engendered a sense of connection and belonging by building on and reinforcing their social capital.

In marginalized groups, individuals who achieve successful boundary crossing can be seen as role models for others (47) and offer opportunities for meaningful social bonding. Nina described how the recovery community offered a ‘*tight bond’* with other women - a bond that allowed Nina to *‘speak the same language’* and express herself in ways she could not with her family:

*“It’s quite refreshing to have a group of women who are all there to watch each other’s backs … I can understand how she’s feeling and why she’s feeling that and well you know there’s a connection there”* (Nina, age 55, recovery 12½ years).

Unable to talk with her family about her past and having moved back to Scotland after two decades away, the bonds Nina developed with the other women were built on shared understandings and a sense of protection which were important in helping develop trust within the group. Nevertheless, being able to talk about their past in a safe and non-judgmental environment is just one element of developing connections and a sense of belonging. Connecting and bonding with others is also about ‘*being heard’*, knowing and feeling that your views are listened to and regarded by others. It is the reciprocity of human connection that is essential for engendering a sense of connectedness and belonging (48):

*“Just being heard has built that esteem and that value. That is one of the most powerful things that I’ve experienced. Being heard”* (Janine, age 47, recovery 21 years).

Making connections leads to a process of bonding and ultimately to a sense of belonging to a ‘*tribe’*. In addition, connecting, bonding, and belonging with others in recovery communities helped some women develop skills and increase their feelings of self-worth.

## Discussion

Connecting and belonging are active processes that are relational, negotiated and performative; processes that people do through their social interactions (16). Individuals must weigh the advantages and disadvantages of connecting and belonging as creating opportunities or restrictions on ways of being. This is illustrated in the women’s interviews regarding their move out of drug use, their connections to families and their engagement with recovery communities. Similar to findings from other studies (14, 24, 49), modifying and transforming relationships were common among the women. Building or re-building positive relationships and protecting themselves from negative relationships are important elements of women’s recovery from substance use (26). In changing their lives and moving from drug use to recovery, the women in this study lost, gained and rebuilt relationships along the way. Their sense of self was constructed in their interactions with others and in the idea of commonly shared social values, standards, and conventions. Belonging also required being able to participate in the world. In other words, participation and belonging required acceptance by others.

The findings from this paper supports research conducted with women and older drug users elsewhere. As with Anderson and Levy’s work on older drug users (6), women in this study who experienced periods of social isolation found re-connecting with the wider social world challenging. As described by Grace and Ruth, personal networks including drug using associates may be difficult to relinquish, particularly if they provide social, emotional or practical support (8). Women may feel challenged in their relationships with parents, especially their mothers (11). Relationships with mothers (and other family members) can be positive to women’s recoveries offering much-needed emotional and practical support but in some cases, women may continue to feel stigmatized or pressured by family members (25). The pressures that arise can be both practical in the sense that once women are deemed ‘recovered’ by family members there is an expectation they will fulfil tasks and roles commensurate with gendered expectations. On the other hand, women may feel guilty for not wanting to take on these roles or internalize a sense of shame or guilt at wanting to distance themselves from family influences they consider detrimental to their recovery.

A few women in this study expressed empathy toward their mothers for the sometimes extremely difficult lives their mothers had experienced but at the same time they wanted to develop boundaries and maintain some level of control in their relationships. Research suggests some family members do not have essential knowledge around addictive drug use and recovery which can lead to unrealistic expectations placed on women, especially in early recovery (50). The women’s interviews in this study add another element to Strauss and Falkin’s (11) work by highlighting the emotional ambivalence and guilt that some women may feel in limiting or cutting contact with parents deemed unsupportive. Managing other people’s expectations of them while convincing others of their abstinent identity, requires diplomacy and boundary-setting that some women in recovery might need to learn (27). Family members, including children, may enable recovery or become a relapse risk (50). Creating personal boundaries and limiting contact are effective ways of managing challenging relationships within the family (11, 25, 47) and are also mechanisms by which women can take ownership of their wider social relationships and enhance their sense of agency (22).

The study findings mirror findings from elsewhere (6, 7) that show recovery is an ongoing process in which women set boundaries with others, assume more control over relationships and develop a recovered identity that others can view as authentic, honest, and genuine. Making connections and belonging to social networks unrelated to active drug use encourages women to develop identities and sense of self as women in their own right, rather than women defined by drug use. This study adds to the body of research on women in recovery from drug use by looking at the experiences of an age and gender cohort that remains relatively under-researched, namely women who are in mid-life and older.

The research question asked what influence social relationships have on women’s sense of self as they age into drugs recovery. The answer is not straightforward. The direction of influence during recovery is complicated and the women’s interviews demonstrate it is a symbiotic process in which relationships, identity and recovery are all highly interdependent. This investigation of women in recovery from illicit drug use shows that a period of abstinence and sobriety can give women space to perform the necessary emotional work and adopt the language of self-acceptance that recovery communities and fellowships espouse. This may encourage women to engage in mindful reflection that can lead to improvements in self-esteem which in turn can impact positively on their relationships with others. Positive reinforcement from others through new relationships and activities can help contribute to better self-concepts that help maintain recovery; one reinforcing the other in a constant interaction. Distancing themselves from a user identity and creating a new abstinent identity is pivotal to the process of women’s self-acceptance. Having the opportunity to connect to others in an actively positive way can counter women’s lack of self-belief brought about by gendered expectations, personal differences and structural and economic inequalities. Moreover, recovery communities can offer opportunities for personal growth and development. They offer space to lead, to take control and to help and support connections to others while also creating a sense of belonging.

Further work around mid-life and older women’s relationships with their parents, and mothers in particular, could aid understanding of family dynamics and how they might help or hinder recovery from drug use. Working with significant others and adult family members in the treatment of women with lived and living experience of drug use using behavioral and systemic approaches may help engage women and improve recovery outcomes (51). As Rowe has argued, attempting to treat people who use drugs outside of the family context may seriously undermine individual recovery (51). Gathering evidence on this issue could help family support and treatment services assess the need for developing support programs and materials for women in mid-life and older age actively using drugs or in recovery.

## Conclusion

This paper explores from women’s standpoints, and within the context of mid-life and older age, their relationships within and out with the family thereby providing greater depth and understanding on a relatively under-researched area in United Kingdom substance use research. In doing so, the paper provides an original and important contribution to studies that seek to understand the intersection of gender, drug use recovery, aging, and social and family relationships.

### Limitations

This was an exploratory study of 19 women in mid-life and older age across the North East of England and Scotland. As with qualitative inquiries in general, the findings of this study cannot be said to represent the relationship experiences of all women with a history of illicit drug use. Utilizing convenience sampling may have resulted in sampling bias therefore speaking with a greater number of women in mid-life and older from across the United Kingdom would elicit a wider range of views and experience while talking to a larger number of women in their fifties and sixties could elicit further information on the impact of aging on women’s family relationships. For example, how do older women navigate and negotiate family expectations around caring for elderly parents? Moreover, including older women from other underserved groups such as women experiencing homelessness, women of color and women in the criminal justice system could add further insights into relationships and recovery among women in mid-life and older age with histories of illicit drug and other substance use. There may have been an opportunity to compare the women’s recovery journeys across different income and education levels and according to sexual orientation, however these demographic details were not sought or provided. Reflecting on the analytical process, two or more coders to allow for inter-rater agreement on a coded transcript would strengthen the analysis. Nevertheless, this study was conducted by an experienced researcher and supervised by experienced and published academics within the field of addictions and additive behaviors.

## Data availability statement

The data that support the findings will be available in University of Glasgow’s repository for research data (Enlighten: Research Data) and the United Kingdom Data Service (University of Essex) following a 12 month embargo from the date of publication to allow for publication of research findings. Requests to access the datasets should be directed to ashaw419@gmail.com.

## Ethics statement

The studies involving human participants were reviewed and approved by University of Glasgow’s College of Social Sciences Research Ethics Committee. The patients/participants provided their written informed consent to participate in this study. Written informed consent was obtained from the individual(s) for the publication of any potentially identifiable images or data included in this article.

## Author contributions

AS was responsible for the conceptualization and design of the study, fieldwork, data management, and analysis.

## Funding

This was a PhD study that was funded by the Economic and Social Research Council (ESRC (DTC) Main ES/J500136/1).

## Conflict of interest

The author declares that the research was conducted in the absence of any commercial or financial relationships that could be construed as a potential conflict of interest.

## Publisher’s note

All claims expressed in this article are solely those of the authors and do not necessarily represent those of their affiliated organizations, or those of the publisher, the editors and the reviewers. Any product that may be evaluated in this article, or claim that may be made by its manufacturer, is not guaranteed or endorsed by the publisher.

## References

[1] Laudet AB. What does recovery mean to you? Lessons from the recovery experience for research and practice. J Subst Abus Treat. (2007) 33:243–56. doi: 10.1016/j.jsat.2007.04.014, PMID: 17889296 PMC2083562

[2] Neale J Nettleton S Pickering L. The everyday Lives of Recovering Heroin Users. London: Royal Society of Arts (2012).

[3] Best D Gow J Knox T Taylor A Groshkova T White W. Mapping the recovery stories of drinkers and drug users in Glasgow: quality of life and its associations with measures of recovery capital. Drug Alcohol Rev. (2012) 31:334–41. doi: 10.1111/j.1465-3362.2011.00321.x, PMID: 21615809

[4] Scottish Government. The Road to Recovery: A New Approach to Tackling Scotland’s Drug Problem. Edinburgh, Scotland: RR Donnelley (2008).

[5] Surrey JL. The "Self-in-Relation": A Theory of Women's Development. Wellesley, MA: Stone Center Working Paper Series (1985).

[6] Anderson TL Levy JA. Marginality among older injectors in today's illicit drug culture: assessing the impact of ageing. Addiction. (2003) 98:761–70. doi: 10.1046/j.1360-0443.2003.00388.x, PMID: 12780364

[7] Bellaert L Van Steenberghe T De Maeyer J Vander Laenen F Vanderplasschen W. Turning points toward drug addiction recovery: contextualizing underlying dynamics of change. Addict Res Theory. (2022) 30:294–303. doi: 10.1080/16066359.2022.2026934

[8] Tracy EM Martin TC. Children's roles in the social networks of women in substance abuse treatment. J Subst Abus Treat. (2007) 32:81–8. doi: 10.1016/j.jsat.2006.06.008, PMID: 17175401

[9] Hamilton AB Grella CE. Gender differences among older heroin users. J Women Aging. (2009) 21:111–24. doi: 10.1080/08952840902837129 19418342 PMC2733531

[10] Jessup MA Ross TB Jones AL Satre DD Weisner CM Chi FW. Significant life events and their impact on alcohol and drug use: a qualitative study. J Psychoactive Drugs. (2014) 46:450–9. doi: 10.1080/02791072.2014.962715, PMID: 25364998 PMC4294766

[11] Strauss SM Falkin GP. Social support systems of women offenders who use drugs: a focus on the mother-daughter relationship. Am J Drug Alcohol Abuse. (2001) 27:65–89. doi: 10.1081/ADA-100103119, PMID: 11373037

[12] Thom B. Women in recovery In: Yates R Malloch M, editors. Tackling Addiction Pathways to Recovery. London, UK and Philadelphia, PA: Jessica Kingsley Publishers (2010). 52–69.

[13] Koenig TL Crisp C. Ethical issues in practice with older women who misuse substances. Subst Use Misuse. (2008) 43:1045–61. doi: 10.1080/10826080801914246, PMID: 18649229

[14] Guerrero M Longan C Cummings C Kassanits J Reilly A Stevens E. Women’s friendships: a basis for individual-level resources and their connection to power and optimism. Humanist Psychol. (2022) 50:360–75. doi: 10.1037/hum0000295, PMID: 36187574 PMC9520295

[15] Kverme B Natvik E Veseth M Moltu C. Moving toward connectedness–a qualitative study of recovery processes for people with borderline personality disorder. Front Psychol. (2019) 10:430. doi: 10.3389/fpsyg.2019.00430, PMID: 30873097 PMC6403141

[16] May V. Self, belonging and social change. Sociology. (2011) 45:363–78. doi: 10.1177/0038038511399624

[17] Allen K-A Kern ML Rozek CS McInerney DM Slavich GM. Belonging: a review of conceptual issues, an integrative framework, and directions for future research. Aust J Psychol. (2021) 73:87–102. doi: 10.1080/00049530.2021.1883409, PMID: 33958811 PMC8095671

[18] McIntosh J. Beating the Dragon: The Recovery from Dependent Drug Use. London: Prentice Hall (2014).

[19] Best D Beckwith M Haslam C Alexander Haslam S Jetten J Mawson E. Overcoming alcohol and other drug addiction as a process of social identity transition: the social identity model of recovery (SIMOR). Addict Res Theory. (2016) 24:111–23. doi: 10.3109/16066359.2015.1075980

[20] Latimer SL. Human connectedness in nursing: a case study. Contemp Nurse. (2013) 44:45–6. doi: 10.5172/conu.2013.44.1.45, PMID: 23721386

[21] Crisp B. Belonging, connectedness and social exclusion. J Soc Incl. (2010) 1:123–32. doi: 10.36251/josi14

[22] Antonsich M. Searching for belonging–an analytical framework. Geogr Compass. (2010) 4:644–59. doi: 10.1111/j.1749-8198.2009.00317.x

[23] Goffman E. The Presentation of Self in Everyday Life. vol. 2002. Garden City, NY: Doubleday and Company, NY (1959). 259.

[24] Francis MW Taylor LH Tracy EM. Choose who’s in your circle: how women’s relationship actions during and following residential treatment help create recovery-oriented networks. J Soc Work Pract Addict. (2020) 20:122–35. doi: 10.1080/1533256X.2020.1748975, PMID: 33414688 PMC7787262

[25] Gunn A Miranda SG. Promoting recovery identities among mothers with histories of addiction: strategies of family engagement. Fam Process. (2020) 59:94–110. doi: 10.1111/famp.12413, PMID: 30556171

[26] Sanders J. Women in Narcotics Anonymous: Overcoming Stigma and Shame. New York, NY: Palgrave MacMillan (2014).

[27] Wangensteen T Hystad J. A comprehensive approach to understanding substance use disorder and recovery: former patients’ experiences and reflections on the recovery process four years after discharge from SUD treatment. J Psychosoc Rehabil Ment Health. (2022) 9:45–54. doi: 10.1007/s40737-021-00233-9, PMID: 34466372 PMC8391873

[28] Collinson B Hall L. The role of social mechanisms of change in women’s addiction recovery trajectories. Drugs Educ Prev Policy. (2021) 28:426–36. doi: 10.1080/09687637.2021.1929077

[29] Matheson C Hamilton E Wallace J Liddell D. Exploring the health and social care needs of older people with a drug problem. Drugs Educ Prev Policy. (2019) 26:493–501. doi: 10.1080/09687637.2018.1490390

[30] Vogt I. Life situations and health of older drug addicts: a literature report. Suchttherapie. (2009) 10:17–24. doi: 10.1055/s-0028-1128135

[31] Bachi K Sierra S Volkow ND Goldstein RZ Alia-Klein N. Is biological aging accelerated in drug addiction? Curr Opin Behav Sci. (2017) 13:34–9. doi: 10.1016/j.cobeha.2016.09.007, PMID: 27774503 PMC5068223

[32] Blumer H. Symbolic Interactionism: Perspective and Method. Berkely and Los Angeles, California: Univ of California Press (1986).

[33] Rolin K. Standpoint theory as a methodology for the study of power relations. Hypatia. (2009) 24:218–26. doi: 10.1111/j.1527-2001.2009.01070.x

[34] Intemann K. 25 years of feminist empiricism and standpoint theory: where are we now? Hypatia. (2010) 25:778–96. doi: 10.1111/j.1527-2001.2010.01138.x

[35] Skinner J. The Interview: An Ethnographic Approach. London, UK: Bloomsbury Publishing Plc (2013).

[36] Oakley A. Interviewing women: a contradiction in terms In: Roberts H, editor. Doing Feminist Research. London: Routledge (2013). 30–61.

[37] Vivancos R Maskrey V Rumball D Harvey I Holland R. Crack/cocaine use in a rural county of England. J Public Health. (2006) 28:96–103. doi: 10.1093/pubmed/fdl010 16648147

[38] Tolland E Kouimtsidis C Reynolds M. Substance misuse among women attending family planning clinics in a rural area in Britain: prevalence and associated problems. Drugs Educ Prev Policy. (2003) 10:195–202. doi: 10.1080/0968763021000057718

[39] Shaw A. Exploring the Influence of Social Relationships on Identity, Drug use and Recovery among Older Drug Using Females. [Master’s Thesis]. Glasgow: University of Glasgow (2017).

[40] Bryman A. Social Research Methods. Oxford, UK: Oxford University Press (2016).

[41] Braun V Clarke V. Using thematic analysis in psychology. Qual Res Psychol. (2006) 3:77–101. doi: 10.1191/1478088706qp063oa

[42] Charmaz K. The search for meanings–grounded theory. In. Smith JA Harre R Langenhove L Van. (Eds.), Rethinking Methods in Psychology (pp. 27–49). London, United Kingdom: Sage (1996).

[43] Bryant A. The grounded theory method In: Leavy P, editor. The Oxford Handbook of Qualitative Research. USA: Oxford University Press (2014).

[44] Carballo JL Fernández-Hermida JR Secades-Villa R García-Rodríguez O. Effectiveness and efficiency of methodology for recruiting participants in natural recovery from alcohol and drug addiction. Addict Res Theory. (2009) 17:80–90. doi: 10.1080/16066350802401295

[45] Perez-Felkner L. Socialization in childhood and adolescence In: DeLamater J and A Ward. (Eds.), Handbook of Social Psychology. New York, NY: Springer Publishing (2013). 119–49.

[46] Pfaff-Czarnecka J. Multiple Belonging and the Challenges to Biographic Navigation. MMG Working Paper 13–05. Max Planck Institute for the Study of Religious and Ethnic Diversity; (2013).

[47] Mahar AL Cobigo V Stuart H. Conceptualizing belonging. Disabil Rehabil. (2013) 35:1026–32. doi: 10.3109/09638288.2012.717584 23020179

[48] Francis MW. Transitions of women’s substance use recovery networks and 12-month sobriety outcomes. Soc Networks. (2020) 63:1–10. doi: 10.1016/j.socnet.2020.04.003, PMID: 32675917 PMC7365593

[49] Boeri M Gardner M Gerken E Ross M Wheeler J. “I don’t know what fun is”: examining the intersection of social capital, social networks, and social recovery. Drugs Alcohol Today. (2016) 16:95–105. doi: 10.1108/DAT-08-2015-0046, PMID: 27668008 PMC5029464

[50] Brown S Tracy EM Jun M Park H Min MO. Personal network recovery enablers and relapse risks for women with substance dependence. Qual Health Res. (2015) 25:371–85. doi: 10.1177/1049732314551055, PMID: 25231945 PMC4608244

[51] Rowe CL. Family therapy for drug abuse: review and updates 2003–2010. J Marital Fam Ther. (2012) 38:59–81. doi: 10.1111/j.1752-0606.2011.00280.x, PMID: 22283381

